# *Dibothriocephalus nihonkaiensis*: an emerging foodborne parasite in Brittany (France)?

**DOI:** 10.1186/s13071-019-3516-6

**Published:** 2019-05-28

**Authors:** Brice Autier, Sorya Belaz, Brigitte Degeilh, Jean-Pierre Gangneux, Florence Robert-Gangneux

**Affiliations:** 1 Univ Rennes, CHU Rennes, Rennes, France; 2 Univ Rennes, CHU Rennes, Inserm, EHESP, Irset (Institut de Recherche en Santé Environnement Travail), UMR_S 1085, 35000 Rennes, France

**Keywords:** Cestoda, Diphyllobothriosis, Foodborne infections, Epidemiology

## Abstract

**Background:**

Diphyllobothriosis is an intestinal cestodosis caused by tapeworms of the family Diphyllobothriidae. In France, endemic cases are limited to south-east and due to *Dibothriocephalus latus*. In this paper, we investigate a series of seven cases of diphyllobothriosis in the non-endemic French region of Brittany. All have been diagnosed between 2016 and 2018 at the University Hospital of Rennes.

**Methods:**

Parasites were identified by their morphological features and by phylogenetic analysis of the *cox*1 gene. Phylogenetic tree was built using maximum likelihood criterion under the GTR+G+I model and 2000 bootstrap replicates. A form was sent to all patients to collect data concerning clinical signs and possible sources of infection.

**Results:**

All cases were due to *Dibothriocephalus nihonkaiensis*, a species strictly distributed in the North Pacific. Epidemiological investigation showed that the parasite was probably acquired in France, after consumption of Japanese food containing raw salmon. All patients presented with at least abdominal pain and fatigue except for one patient who had no symptoms.

**Conclusions:**

To our knowledge, this case series is the most important cohort of allochthonous diphyllobothriosis described in Europe. This sudden emergence raises concern about foodborne infections, highlighting (i) risky food habits in absence of adequate sanitary control; and (ii) the breaking of the rule of geographical restriction due to globalization and worldwide trades.

## Background

Intestinal cestodoses are cosmopolitan foodborne parasitic diseases, responsible for an important morbidity worldwide [1]. Principal agents of human intestinal cestodoses are *Taenia* spp., *Hymenolepis* spp. and *Dibothriocephalus* spp. [1, 2]. Epidemiology of these infections varies greatly amongst areas in the world, in relation to the geographical distribution of the parasites and the local food habits. In France, autochthonous tapeworm infections are due to *T. saginata* and, in the region of Swiss Lakes, to *Dibothriocephalus latus*.

*Dibothriocephalus nihonkaiensis* is an intestinal tapeworm belonging to the family Diphyllobothriidae. Like all other members of this family, its life-cycle involves a first intermediate host, which is a copepod crustacean, harbouring a procercoid larva differentiated from a coracidium previously liberated from the egg. After ingestion of the infected copepod by a specific fish, the larva develops from a procercoid to a plerocercoid stage, localized in various tissues [3]. The definitive host becomes infected by eating raw or undercooked fish containing the plerocercoid larva. In the case of *D. nihonkaiensis*, infection occurs through the consumption of salmon originating from North Pacific. The larva develops rapidly to adult form and first eggs are spread with the stools 2–6 weeks after infection [3].

*Dibothriocephalus nihonkaiensis* cases were mostly described in Asia [4–9]. As recently published, it accounts for a vast majority of intestinal tapeworm infections in Japan, representing 86% of causative agents encountered [4]. However, some cases of *D. nihonkaiensis* infection due to the consumption of imported Pacific salmon have been also reported across the world [5]. In Europe, only six cases have been reported since 2005, although additional cases have been probably misdiagnosed or not reported [10, 11]. In France, endemic *D. latus* infection is restricted to the south-east, and diphyllobothriosis with other species than *D. latus* is only due to imported fish and exceptional. Indeed, only two cases of infection with *D. nihonkaiensis* were reported in 2005 and 2008, due to imported Pacific salmon purchased in France, and no other species have been reported until now [12, 13]. In this study, we investigated seven consecutive cases of diphyllobothriosis due to *D. nihonkaiensis*, diagnosed from 2016 to 2018, in the non-endemic region of Brittany (western France).

## Methods

### Patients and parasite specimens

Specimens were brought by patients at the consultation unit of the Laboratory of Parasitology and Mycology of the Rennes University Hospital, or collected by clinical laboratories in the east of Brittany and sent to our laboratory for identification. All cases were diagnosed between July 2016 and September 2018. Diagnosis of infection by a tapeworm belonging to Diphyllobothriidae was performed by morphological analysis of strobilae of worms spontaneously emitted by the patient (6 cases). In the remaining case, the diagnosis was made on the observation of eggs during direct microscopic examination of stools, as part of the usual laboratory practice. A form was sent to all patients to collect data on clinical signs and possible sources of infection.

### DNA extraction

DNA were extracted from proglottids of patients #2 to #7 using the QIAmp Mini Kit (QIAgen, Courtaboeuf, France), following the manufacturer’s recommendations. Because no adult worm has been obtained for patient #1, DNA was extracted from eggs in the stools using the QIAmp Stool Mini Kit (QIAgen, Courtaboeuf, France). Some modifications of the recommended protocol were applied: (i) stool was incubated in proteinase K and lysis buffer overnight instead of 10 min; and (ii) after the enzymatic lysis a bead-beating step was added, with the MagNA Lyser device (Roche Diagnostics, Meylan, France) and the MagNA Lyser Green Beads (Roche Diagnostics, Meylan, France) at full speed for 35 s.

### Sequencing analysis

Sequencing analysis was performed on a 698 bp fragment of the cytochrome *c* oxidase subunit 1 (*cox*1) gene amplified with the specific primers JB6 (5′-GAT AGT AAG GGT GTT GA-3′) and JB5R (5′-CAA GTA TCR TGC AAA ATA TTA TCA AG-3′) described by Yera et al. in 2008 [14]. PCR mixes contained 1 U of HotStarTaq^®^ DNA polymerase (QIAgen, Courtaboeuf, France), 1.5 mM of MgCl_2_, 200 nM of each primer and 200 µM of dNTP, in a final volume of 50 µl including 10 µl of DNA template. For each PCR reaction a negative control (sterile water instead of DNA template) was included. Amplification was realized with the Veriti^®^ Thermal Cycler (Applied Biosystems, Courtaboeuf, France). PCR was carried out through 45 cycles of 30 s at 95 °C, 40 s at 50 °C and 1 min at 72 °C. Length and specificity of PCR products were checked by migration in a FlashGel^®^ system (Lonza, Rockland, USA). After purification of PCR products with polyacrylamide gel (Bio-Gel^®^ P-100, Bio-Rad^®^, Marnes-La-Coquette, France), sequencing reactions were performed using the BigDye Terminator v3.1 kit (Applied Biosystems), according to the manufacturer’s instructions. The sequencing products were then purified with the BigDye XTerminator^®^ purification kit (Applied Biosystems) before running on an Abi Prism 3130 Genetic Analyser (Applied Biosystems). After validation of the sequences using Sequence Analysis Software v5.4 (Thermo Fisher Scientific, Dardilly, France), these were submitted to Blast Search (https://blast.ncbi.nlm.nih.gov/Blast.cgi). Partial sequences (603 bp) of the *cox*1 gene were aligned with sequences retrieved from GenBank for *D. nihonkaiensis*, *D. latus*, *D. dendriticus* and *D. ursi* using ClustalW [15] implemented in MEGA v.6 [16]. Phylogenetic analysis was run under the maximum likelihood criterion in MEGA v.6 [15], under the GTR+G+I model and bootstrap resampling of 2000 replicates. The sequences of the *cox*1 gene of *Ligula intestinalis* and *Diphyllobothrium balaenopterae*, two species belonging to the family Diphyllobothriidae and close to the genus *Dibothriocephalus*, were used as outgroups. The newly generated sequences were submitted to the GenBank database under the accession numbers MK070860-MK070866.

## Results

### Clinical and biological characteristics

Patients were 16 to 48 years-old (median of 30 years) and the male:female sex ratio was 1.3:1. They presented willingly at medical consultation because of clinical signs or worm emission (strobila length varying between 10 cm and 4 m). All patients presented with at least abdominal pain and fatigue except for one patient who had no symptoms. Five out of seven had diarrhea, while patient #3 had weight loss (3 kg) and itch, and patient #7 had nausea. Hemoglobin level was measured for patients #1, #4, #5 and #7, and B12 vitamin was measured for patients #4 and #5. Results were normal for all of them except for patient #1 who had anemia; however, this patient was a pregnant female and anemia was more probably due to pregnancy (normal mean corpuscular volume).

**Fig. 1:**
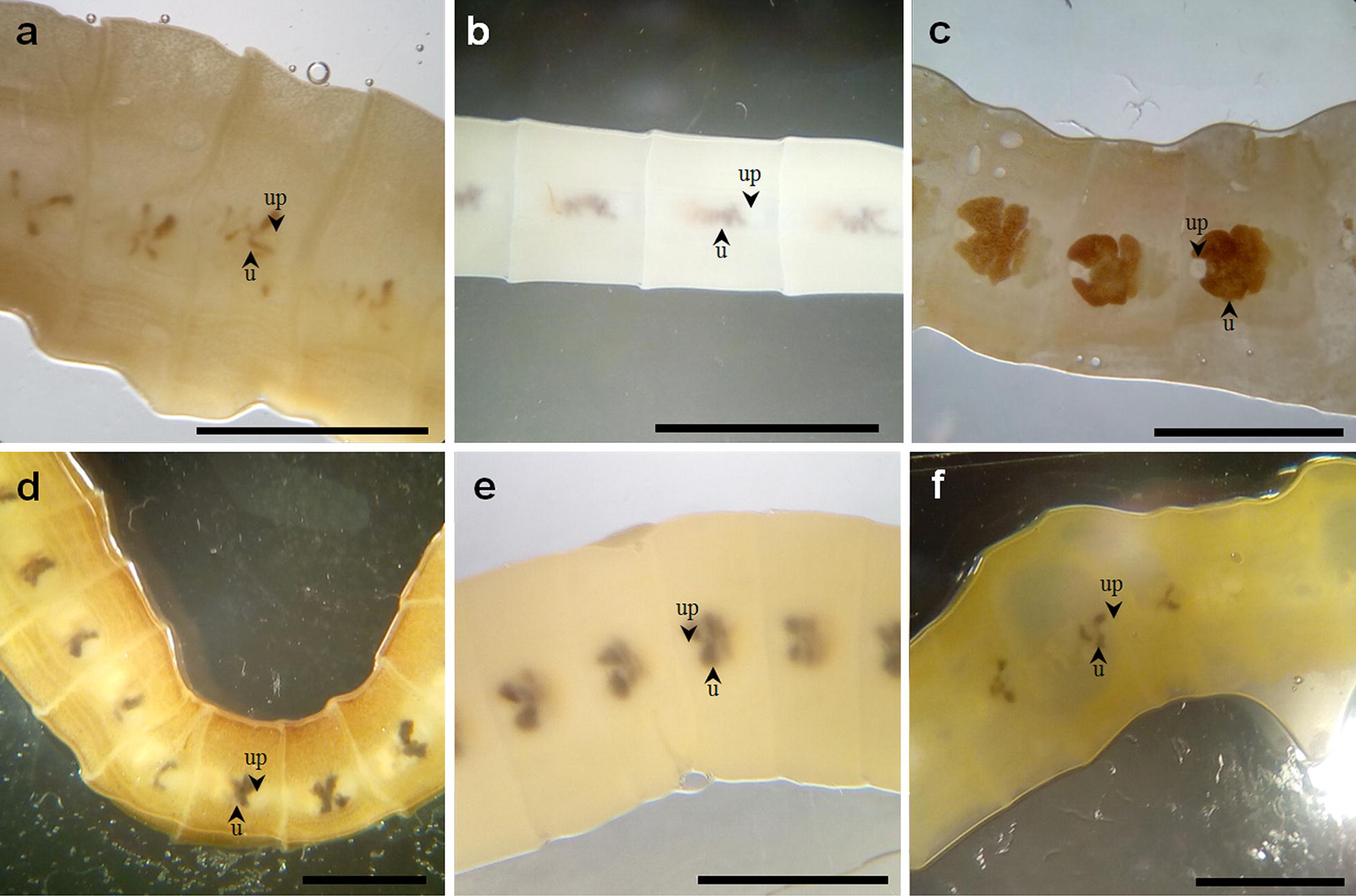
Morphology of the diphyllobothriid tapeworm specimens collected in Brittany. **a**–**f** Patients #2 to #7. Central uterine pore (up) and rosette-shaped uterus (u) are indicated by arrowheads. *Scale-bars*: 5 mm

Strobilae were collected for all patients except for patient #1, and specimens presented similar morphological characteristics compatible with tapeworms belonging to the Diphyllobothriidae such as trapezoidal proglottids (3 mm × 5–10 mm) with a central uterine pore and a rosette-shaped uterus (Fig. 1). In all cases, eggs were ovoid, operculate, with a thin, slightly tainted shell. They measured 40–50 µm in width and 55–66 µm in length.

The main characteristics of each case, including putative source and area of infection, are detailed in Table 1. Consumption of farmed smoked salmon, which is unlikely at risk for infection, has not been mentioned. Travels outside France have been cited only when associated with raw fish consumption.

**Table 1:** Characteristics of the seven patients with *D. nihonkaiensis* infection diagnosed in Brittany (France) from 2016 to 2018

Patient #	Sex	Age (years)	Date of diagnosis	Putative source of infection	Putative area of infection	Clinical signs	Observed stage	Additional data	GenBank ID
1	F	27	July 2016	Japanese food	France	Abdominal pain, fatigue, diarrhea	Eggs		MK070861
2	M	16	September 2016	Smoked salmon or Japanese food	Probably France, possibly Sweden or Norway	None	Adult	Ate traditional smoked salmon in Norway (2004, 2007) and Sweden (2011)^a^	MK070860
3	F	48	August 2017	Japanese food	France	Abdominal pain, fatigue, diarrhea, loss of weight, itch	Adult	Ate raw fishes in Asia but only freshwater fishes	MK070862
4	M	30	January 2018	Japanese food	France	Abdominal pain, fatigue, diarrhea	Adult		MK070863
5	M	41	July 2018	Japanese food	Probably France, possibly border country	Abdominal pain, fatigue, diarrhea	Adult	Microscopic examinations of stools in October 2017 were negative for parasites^b^	MK070864
6	M	42	August 2018	Undercooked salmon or Japanese food	France	Abdominal pain, fatigue, diarrhea	Adult	In early 2018: consumed undercooked salmon of unknown origin, bought at a local supermarket in Corrèze (France)	MK070865
7	F	20	September 2018	Japanese food	France	Abdominal pain, fatigue, nausea	Adult		MK070866

^a^As traditional smoked salmon is commonly made with Atlantic salmon in Northern Europe and *D. nihonkaiensis* is acquired through ingestion of Pacific salmon, contamination probably occurred by sushi consumption. However, the use of infected Pacific salmon by a smokery could not be completely excluded

^b^This patient was previously followed for pericarditis associated with 3 G/L hypereosinophilia and positive serology for anisakiosis (October 2017). After exclusion of other possible etiologies, a treatment with corticosteroids and albendazole was administered in April 2018

### Phylogenetic analysis

**Fig. 2:**
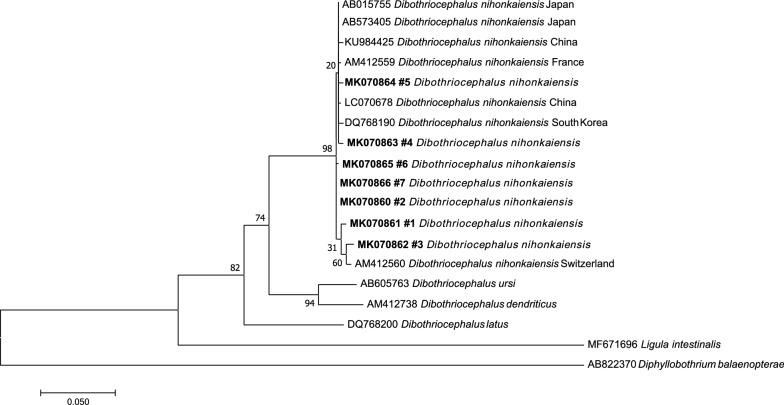
Phylogenetic relationships between clinical isolates from Brittany, *D. nihonkaiensis* specimens isolated from different countries and other species of *Dibothriocephalus*, based on a partial sequence of the *cox1* gene. Nodal values are bootstrap values expressed in percentages out of 2000 replicates. *Ligula intestinalis* (MF671696) and *Diphyllobothrium balaenopterae* (AB822370) were used as outgroups. The newly generated sequences are shown in bold

For each specimen, sequence analysis of the *cox*1 gene allowed the identification of *D. nihonkaiensis* as the causative agent (≥99% pairwise sequence identity). The phylogenetic analysis clearly resolved a monophyletic lineage consisting of all specimens of *D. nihonkaiensis* reported here (MK070860 to MK070866 for patients #1 to #7, see assignment detailed in Table 1) and the representative sequences for *D. nihonkaiensis* included in the analysis (Fig. 2). The interrelationships within this lineage remained unresolved, with the newly reported sequences failing to form an independent lineage.

## Discussion

In this study, we report a case series of seven consecutive cases of infection by *D. nihonkaiensis*, all diagnosed in patients from eastern Brittany, France. Almost all patients presented with mild and non-specific clinical signs. Our epidemiological investigation suggests that these infections had been likely acquired in France (or at least in Europe for patients #2 and #5). All of them were probably due to sushi consumption, even if another source of infection could not be excluded for patients #2 and #6. No Japanese restaurant commonly frequented by several patients could be identified. Classical parasitological methods allowed the diagnosis of infection due to a tapeworm of the family Diphyllobothriidae, but sequencing of a diagnostic gene was necessary to identify the specimens as *D. nihonkaiensis*. Our phylogenetic analysis failed to resolve if isolates belonged to the same intraspecific lineage, but the well-conserved marker *cox*1 is not adapted to differentiate individual populations [2, 14, 17].

This is the most important series of allochthonous infections with tapeworms from the family Diphyllobothriidae in Europe. Indeed, until this report, only 17 cases have been described in Europe over 13 years, including 6 cases of *D. nihonkaiensis* [10], four cases of *D. dendriticus* [10, 18], five cases of *A. pacificus* [19, 20] and two cases of *Diphyllobothrium balaenopterae* [20]. The cases reported here occurred in a surprisingly short period (two years), whereas no other cases had been diagnosed during the past 20 years at our institution. Moreover, as there is no French national reference center for intestinal cestodosis, diphyllobothriosis is not reported by medical practitioners, implying that other cases could have been missed. This suggests a shift in the incidence of allochthonous diphyllobothriosis, which is probably underestimated, as it can be misidentified as *D. latus* [10, 20].

In an epidemiological study on agents of cestodosis in Japan from 2001 to 2016, Ikuno et al. [4] concluded that *D. nihonkaiensis* infection was predominant (86% of cases) and that its incidence might increase worldwide because of marketing globalization. The present observations strongly support this conclusion, and highlights that diphyllobothriosis is no more area-restricted, as previously described. The underlying explanation is that fishes on the market are transported from their fishing grounds to the selling locations on ice without freezing, which allows the parasites to survive [3]. Imported parasite species may vary according to trading exchanges between countries. For example, in Spain, where fishes are often imported from South America, allochthonous infection by fishborne tapeworms seems to be mainly due to *A*. *pacificus*, a species encountered off the Pacific coast of South America and transmitted through consumption of marine fishes [19]. Consequently, molecular identification is needed, otherwise major epidemiologic changes might be missed.

Diphyllobothriosis has a moderate impact on human health. Infections are often asymptomatic until proglottids are expelled with stools. For symptomatic people, the main clinical signs are fatigue and gastrointestinal troubles like abdominal pain and diarrhea [3]. Historically in highly endemic areas, complications such as megaloblastic anemia and cholangitis have been observed, but they are now rarely encountered [3]. Nevertheless, such an increase in the occurrence of diphyllobothriosis probably testify to the lack of compliance with the European recommendations for food workers (freezing of raw fish at temperatures lower than −20 °C for at least 24 hours; regulation N° 853/2004 of the European parliament), as well as changes in food habits, with increasing consumption of sushi and raw fish in France, possibly combined with a poor knowledge of customers regarding prevention.

## Conclusions

This sudden series of cases of *D. nihonkaiensis* infection reminds that globalization is an active contributor to the spread of infectious diseases through worldwide trade, but also through adoption of food habits. If sanitary surveillance does not anticipate this phenomenon, rapid modifications in the distribution of parasitic diseases could occur, with possible hazardous consequences. First efforts should aim at implementing a more active surveillance of raw fish products and at providing prevention information to consumers.

## Data Availability

Data supporting the conclusions of this article are included within the article. The newly generated sequences were submitted to the GenBank database under the accession numbers MK070860-MK070866.
